# Research on Energy and Economics of Self-Made Catalyst-Coated Membrane for Fuel Cell under Different Oxidants

**DOI:** 10.3390/membranes12020128

**Published:** 2022-01-21

**Authors:** Qiang Bai, Chuangyu Hsieh, Shaobo Li

**Affiliations:** 1 School of Mechanical Engineering, Guizhou University, Guiyang 550025, China; 2 Department of Automotive Engineering, Tsinghua University, Beijing 100084, China; heyx19@mails.tsinghua.edu.cn; 3 State Key Laboratory of Public Big Data, Guizhou University, Guiyang 550025, China; lishaobo@gzu.edu.cn

**Keywords:** PEMFC, oxygen, ion-exchange membrane, polymer membrane, economics

## Abstract

In the context of global warming, clean energy represented by fuel cells has ushered in a window period of rapid development; however, most research mainly focuses on the improvement of catalysts and performance, and there is very little research on the performance differences and energy consumption between different oxidants. In this paper, the performance differences of fuel cells with different oxidants (air and oxygen) are studied using a self-made CCM, and the economic aspect is calculated from the perspective of power improvement and energy consumption. Firstly, the CCM and GDL are prepared, and the hydrophilicity and hydrophobicity of GDL are realized by the addition of PTFE and SiO_2_, respectively. Secondly, through the experiment, it is found that the fuel cell can achieve the best comprehensive performance at 60 °C, and the use of oxygen can achieve the highest power increase, 117.1%, compared with air. Finally, from the perspective of economics, after excluding the power consumed for preparing oxygen, the use of oxygen as an oxidant still achieved a net power increase of 29.512%. The research in this paper clearly shows that using oxygen instead of air can greatly improve performance and is good economically, which makes it a useful exploration for the research of fuel cells.

## 1. Introduction

As a very promising new energy research field, proton exchange membrane fuel cells (PEMFC) have great advantages in energy conversion efficiency and environmental protection. Especially given the background of global energy conservation and emission reduction, “carbon peak” and “carbon neutralization”, countries all over the world have invested many human and material resources to study fuel cells. Lithium batteries are a widely used new energy technology, but they have several disadvantages, as follows: they have a long charging time; the performance deteriorates seriously after long-term use; potential for explosions, caused by overheating or diaphragm damage. Therefore, the increasingly mature fuel cell technology will be gradually applied to the fields of transportation and distributed generation, and will gradually become the best solution in the field of new energy. In distributed generation, the cost-effective redox flow batteries proposed by NASA in the 1970s are also a promising research direction [1,2]. However, due to the problems of high initial investment and low energy density, it is necessary to continue to study this technology to improve its commercial application value. At present, the research on PEMFC mainly focuses on performance improvement, power system design and materials economics [3,4,5,6,7,8], and less on oxidants.

Polymer membranes composed of MEA and GDL are the core structure of fuel cells and play a decisive role in cell performance, and this is also a key research field of global research [9,10,11,12,13,14]. Fabrizia Foglia [10] et al., studied the dynamic process of ion conduction in polymer membranes, discussed the serial decoupling approach, and improved the performance of MEA through system optimization. Zimo Wang [11] et al., improved the comprehensive performance of MEA (the dispersion, mechanical properties, proton conductivities, swelling ratios, and water uptake), through modification and functionalization of the metal organic frameworks (MOFs) in polymer membranes. In Seop Lim [12] et al., in order to make fuel cells achieve an excellent performance, under high humidity and low humidity, HfO2 was deposited on the MPL side of conventional reference GDL, using atomic layer deposition (ALD). The experimental results show that adding an appropriate amount of HfO2 can not only improve the performance of the fuel cell under low humidity, but also improve the performance of the fuel cell under high humidity. Zahra Rajabi [13] et al., found that the use of PAMAM mesoporous can be a good option to improve the structural and electrochemical properties of the PPBI/ Mim7 þ membranes, for use in the elevated temperature PEM fuel cells in long-term operations. The above research indicates that the existing studies focus on the improvement of MEA materials and structure, and then compare with the existing MEA performance, but there are few comprehensive studies on oxidizers and economics.

Oxidants are the basic reaction elements of fuel cells, and concentration of oxygen and its distribution in the runner plate will have a great impact on the performance of fuel cells. At present, the oxidant is generally air, but the oxygen content in the air is low, which limits the improvement of fuel cells, and other components in the air (CH4, CO2 and CO) will damage PEMFC. Therefore, some researchers have used oxygen as an oxidant to improve the performance of the PEMFC, while ensuring the quality and stability of the oxidant [15,16,17,18,19]. Lixin Fan [15] et al., designed a pressure-based purging device in the pure oxygen fuel cell power system to improve the utilization of oxygen and hydrogen. Meiling Deng [16] et al., studied the performance difference of the PEMFC under different concentrations of oxygen, at a high temperature, and made a beneficial exploration for the adjustment of oxidants. Liangzhen Yin [17] et al., tested the performance difference of PEMFC under anoxic and oxygen saturation conditions, which accumulated a research basis for improving system efficiency. Vrushali M. Umap [18] et al., studied the optimal platinum load under different oxidants (air and oxygen), which contributed to reducing the use of platinum and the cost of PEMFC. Pramod Behari Lal chaurasia [19] et al., designed a hybrid power generation system, combining solar energy and fuel cells, and tested the performance of fuel cells with different proportions of a liquid oxidant mixture, and the H_2_ comes from the dehydrogenation reactor. The above research mainly focuses on optimization, with the power system as the core objective, and the fuel cells used are commercial products. In this paper, self-made polymeric membranes and assembled fuel cells will be used to study the performance differences between different oxidants.

Economics has always been one of the difficulties with regard to fuel cells, hindering their wide application. Xi Chen [20] et al., constructed a methanol reforming PEMFC power generation system with a geothermal energy source, and the levelized cost is 0.0422 USD/kWh. Francesco Calise [21] et al., designed a polygeneration system with fuel cells and solar energy at its core, to provide clean energy for small buildings, and it is expected that it will be ready within five years. Zhen Wu [22] et al., designed a hybrid fuel cell power generation system with natural gas as the energy, in which SOFC and PEMFC account for 60% and 20% of the total cost, respectively, and the power generation cost in ten years is 0.04058 USD/kWh. The above economic research is generally conducted from the perspective of finance or power system cost. Although they are all denominated in US dollars, they have poor universality, which is due to different subsidy policies for clean energy all over the world. Developed countries have developed economies, so they have higher subsidies for clean energy, while developing countries have relatively few subsidies. Therefore, this paper will research the economics of fuel cells with different oxidants, from the perspective of power consumption. Based on the above analysis, the effects of different oxidants on the performance of fuel cells under different temperatures will be researched, and the economics from the perspective of power improvement will be calculated, which will be a beneficial exploration for the further development of fuel cells.

As mentioned above, this paper systematically studied PEMFC from the following three aspects: MEA production, a performance test of different oxidants at different temperatures and economic analysis. Firstly, the hydrophobic and hydrophilic GDL are prepared and the catalyst layer is prepared by the CCM method. Secondly, the performance difference of the fuel cells under two different oxidants, air and oxygen, is studied. It is found that the use of oxygen greatly improved the performance. Finally, the fuel cell economy is calculated from the perspective of energy consumption and power increase, and the calculation results show that the use of oxygen achieves the net increase in power and has good application value.

## 2. Materials and Methods

### 2.1. Material

**Figure 1:**
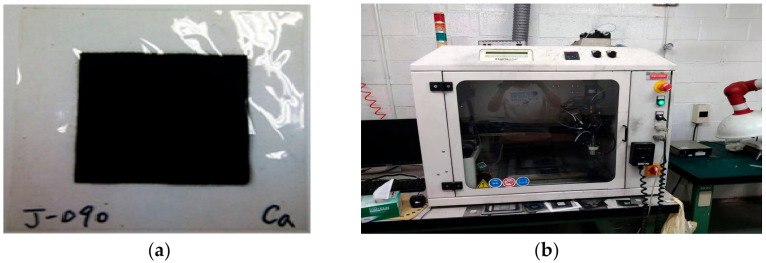
CMM and automatic spraying machine. (**a**) Self-made CCM. (**b**) Automatic spraying machine.

Membrane electrode assembly (MEA) is the core component of PEMFC, which greatly affects the performance and service life of cells. Therefore, a significant amount of research has been conducted on it, as well as how to improve it [23,24,25,26]. Nafion is the base of MEA and, due to the hydrophobic fluorocarbon backbones of Nafion, it has excellent thermal and chemical stability and owing to the -SO_3_H as the hydrophilic domains, the membrane absorbs water easily. Furthermore, the phase segregation between hydrophilic and hydrophobic domains leads to the proton-conducting channels, hence, Nafion is widely used on PEMFC and redox flow battery [27]. However, Nafion membrane also faces the challenges of high cost and competition from several low cost sulfated polymer membranes (such as SPEEK membranes and organic membranes, etc.). Therefore, technical improvement is needed to consolidate the competitiveness of Nafion membranes [28].The MEA structure in this paper is mainly divided into the following two parts: gas diffusion layer (GDL) and catalyst-coated membrane (CCM). GDL is a multi-space structure, which diffuses the H_2 –_O_2_ inside the PEMFC and then evenly distributes it on CCM; CCM essentially sprays catalyst (Pt/C) on proton exchange membrane to make oxidant and reductant react. At present, the mainstream preparation methods of membrane electrode assembly are CCS (catalyst-coated substrate) and CCM (catalyst-coated membrane). CCS is the mainstream method for early preparation of the MEA, which has advantages of simple method and high yield, but it has disadvantages of high interfacial impedance between catalyst layer and membrane material, and ultra-low platinum loading via sputtering technique will reduce the service life of MEA [29,30]. Decal transfer is the mainstream method of CCM at present, and it is also the method used to prepare the MEA in this study. Specifically, the catalyst is sprayed on the transfer substrate, and then the catalyst layer is transferred on both sides of the MEA through hot pressing process, and the amount of catalyst of PEMFC used in this article is 0.35 mg/cm^2^. This method can reduce the interface impedance and the finished product is shown in Figure 1a. Figure 1b shows the Sono-Tek automatic spraying machine used in this study, and the electrolyte membrane is Nafion^®^-HP, the catalyst is 20 wt% Pt/C. In order to overcome the problem of wet expansion during spraying, the spraying process needs to be carried out slowly. The specific steps are as follows:

(1) Mix the catalyst with 5 wt% Nafion solution, then add a mixed solution of isopropanol solvent and deionized water, wherein the content ratio of catalyst to Nafion is 2:1, and place it into the ultrasonic oscillator for two hours to fully mix the solvent;
(2) Clean the pipeline of the automatic spraying machine with isopropanol, open the air pressure bottle, and set the temperature of the heating plate to 80 °C;
(3) After reaching the specified temperature, place the solution in (1) in the pipeline of the automatic spraying machine and conduct positioning test to determine whether the solution spraying can be carried out normally;
(4) Set spraying parameters and conduct path test to determine whether the spraying position is correct. After confirmation, membrane spraying can be carried out;
(5) Place the sprayed membrane in the oven and bake it at 60 °C for 8 h, then use shell protection paper for hot pressing protection (1000 psi-130 °C-3 min), and finally complete the production of the CCM.

**Figure 2:**
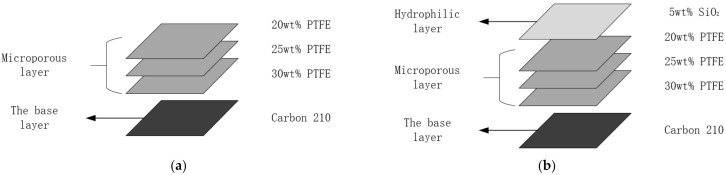
The structure of GDL. (**a**) The structure of cathode-hydrophobicity. (**b**) The structure of anode-hydrophilicity.

The fabrication of GDL is mainly composed of hydrophilic treatment and spraying of microporous layer, which gives it the ability of water management and gas diffusion at the same time, so as to improve the performance of the PEMFC. The hydrophilic layer is based on the hydrophilicity of SiO_2_, which is added to the membrane surface to realize water management, and the microporous layer is to spray dense conductive carbon powder on the membrane surface to improve the gas diffusion performance. In order to solve the problem that the single-layer microporous layer is prone to membrane rupture due to uneven stress, three layers of microporous layer are sprayed in this study and the overall structure is shown in Figure 2. The specific manufacturing process is as follows:

(1) Use a high-precision balance to measure the quality of carbon paper;
(2) Mix carbon powder with isopropanol and place it into the ultrasonic oscillator for two hours to fully mix them;
(3) In order to improve the hydrophobicity of the cathode, during the shaking process, PTFE (Teflon) is slowly added and the process is to use the mixed solution of carbon powder and PTFE as the hydrophobic layer material;
(4) In order to improve the hydrophilicity of anode end, during the shaking process, SiO_2_ powder is slowly added and the process is to use the mixed solution of carbon powder and SiO_2_ as the hydrophilic layer material;
(5) Spraying the solution prepared in (3) and (4) onto the surface of carbon paper with a spraying machine;
(6) The sprayed GDL is placed in a high-temperature furnace for sintering, and the temperature is set as: (120 °C, 30 min), (280 °C, 30 min), (390 °C, 30 min).

After the MEA is fabricated, it needs to be assembled inside the fuel cell to test the performance of cell. The cell assembly process is as follows:

(1) Wipe the bipolar plate and graphite plate with industrial alcohol and check the surface flatness;
(2) Stack the end plate with the red copper gold-plated collector plate, put the bolts into the fixing holes in sequence, and then sleeve the Teflon tube on the outer surface of the bolt, so as to fix the bolt position and prevent the anode and cathode of the PEMFC from conducting with each other;
(3) Place the runner graphite plate on the bipolar plate, and then sleeve the MEA into the bolt;
(4) Install the other side of the PEMFC according to the sequence in (1–3);
(5) Tighten the PEMFC with a torque wrench in a diagonal locking manner. In order to avoid deformation of the MEA caused by stress concentration in the PEMFC, apply a force of 5 kg each time and gradually increase the locking force to 25 kg.

### 2.2. Method

The assembled PEMFC also needs to be tested to ensure its normal operation, and the test is mainly divided into air leakage inspection and MEA activation. Air leakage will seriously affect the performance of PEMFC and have great potential safety hazards, which can be divided into the two following situations: outside PEMFC leakage, which is mainly caused by installation deviation during assembly or material problems, and air leakage, which is similar to the former, except that the air leakage position is inside the PEMFC. In this study, a floating ball flowmeter and air leakage tester are used to realize the preliminary selection and accurate analysis of the air leakage problem respectively. MEA activation is an important step before the PEMFC works normally, and the specific process is as follows:

(1) Install the PEMFC on testing platform and complete the parameter setting;
(2) Supply oxidant and reductant and maintain the open circuit voltage for 10 min. The next step can be carried out after the state is stable;
(3) Set the working voltage of single PEMFC to 0.6 V and maintain for 30 min, then adjust the voltage to 0.2 V and maintain 30 min, cycle 12 times, a total of 12 h;
(4) Observe whether the polarization curve of the PEMFC in (3) is stable and whether the performance meets the standard, and then the next test can be carried out.

**Figure 3:**
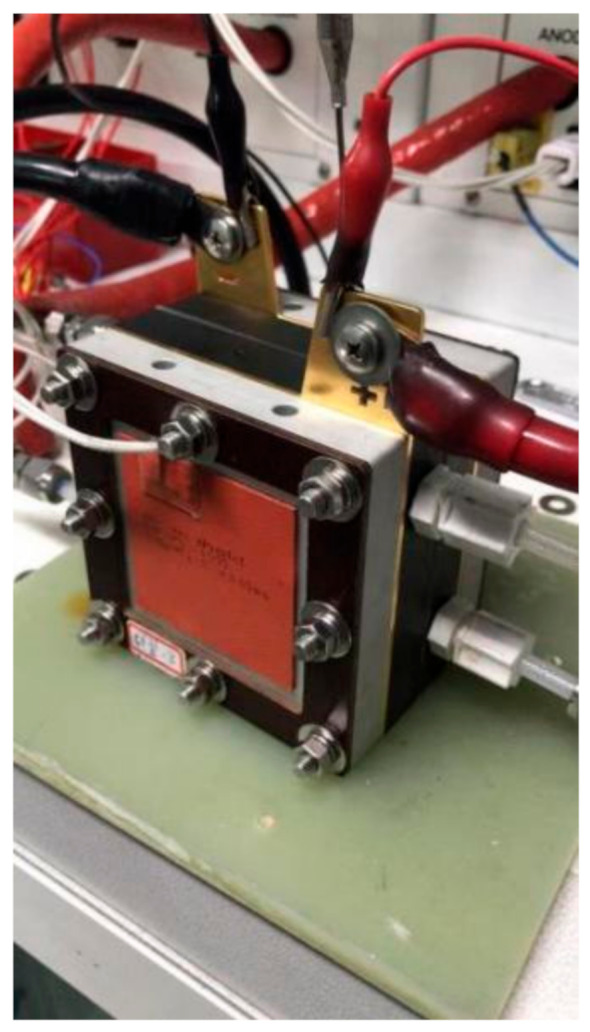
Overall structure of PEMFC.

After MEA preparation and cell assembly test, the performance of the PEMFC meets the requirements, and the overall structure is shown in Figure 3. The reaction area of proton exchange membrane used in this study is 10 cm^2^, and the number is 2 cells.

### 2.3. The Stoichiometry of Oxidant and Reductant

At present, the oxidant commonly used in PEMFC is air. This is because air is easy to use, but it also has disadvantages of many impurities and low oxygen content. Therefore, this paper set out to research the performance differences between different oxidants. Firstly, this paper uses air as the reference oxidant, initially input 2 times stoich and increase 0.2 times to 3 times in turn. Secondly, oxygen is used as oxidant. The use of oxygen not only improves the content of oxygen, but also saves the steps of air filtration, keeps the whole working process of the PEMFC in a closed space, improves the control of the overall environment, and the input stoich is the same as that of air. Finally, the initial input stoich of hydrogen is 1 time and increases 0.2 times to 2 times in turn.

## 3. Results

At present, PEMFC are mainly divided into the following two development trends: high temperature (above 150 °C) and low temperature (below 100 °C). Although high-temperature fuel cells can improve performance and tolerance to toxic substances, they also reduce cell life to a great extent. Low-temperature fuel cells have better durability and safety, and their overall structure is also relatively simple. At present, the research scope of low-temperature fuel cells is mostly concentrated in the range of 50–80 °C [18,19,31,32,33]. We conducted a rough test of the fuel cell performance at different temperatures (30–100 °C), and found that the comprehensive performance of the fuel cell is best at 50 °C–60 °C–70 °C. Therefore, this paper used three conditions of 50 °C, 60 °C and 70 °C to test the performance of the PEMFC.

### 3.1. Working Principle of PEMFC

The working principle of PEMFC is equivalent to the reverse reaction of electrolytic water, and a single cell is composed of an anode, cathode and proton exchange membrane. The anode and cathode are, respectively, hydrogen and oxygen for the redox reaction, and the proton exchange membrane, as the medium for transmitting hydrogen ions, only allows hydrogen ions to pass through, while the electrons lost by hydrogen pass through the wire to generate a current. The basic chemical reaction equation of PEMFC is follows:

Anode: H_2_ → 2H^+^ +2e^−^
Cathode: 1/2O_2_ + 2H^+^ +2e^−^ → H_2_O
Overall: H_2_ + 1/2O_2_ → H_2_O + heat (1)

The heat generated by hydrogen combustion (Enthalpy, ΔH) is 286 kJ mol^−1^ (25 °C). From the perspective of the electrochemical reaction of fuel cells, their chemical energy is converted into electrical energy and thermal energy, and the upper limit of the electrical energy is 237.34 kj mol^−1^ Gibbs free energy (Δ*G*); the thermal energy *T*Δ*S* that must be lost in the conversion process is 48.68 kj mol^−1^. The electric energy thermodynamic equation of the electrochemical reaction of hydrogen after passing through the fuel cell is as follows:

(2) ΔG=ΔH−TΔS

The electric energy generated by the PEMFC includes charge and potential, seen in the following equation:

(3) Wel=qE=nFE

where *q* is the electric quantity, *E* is the potential, *Wel* is the work done by the PEMFC, *F* is the Faraday constant, and *n* is the number of electrons per unit.

In conclusion, the maximum electrical energy generated by the PEMFC is Gibbs free energy (Δ*G*), so the theoretical potential of the fuel cell can be derived from the following formulas:

(4) Wel=nFE=ΔG

(5) E=ΔG/nF

Since Δ*G*, *n* and *F* are known values, the unit theoretical potential of PEMFC is calculated as follows (25 °C, 1 atm):

(6) $$E=\frac{\Delta G}{nF}=\frac{237.34\;Jmol^{-1}}{2\times 96.485\;Asmol^{-1}}=1.23\;Volts$$

According to the above formula, the theoretical potential of the PEMFC is 1.23 V, but the actual potential change is due to the changes of its operating temperature, pressure and concentration. Therefore, 1.23 V is generally referred to as the theoretical potential of hydrogen PEMFC. From the perspective of application, high voltage will lead to an unstable performance and low current, which will also reduce power. Therefore, the working voltage of commercial fuel cells is generally 0.6–0.65 V, and in this study, 0.6 V is used as the working voltage of a single PEMFC.

### 3.2. Differences in PEMFC Performance at Different Temperatures—Air

A PEMFC is a multi-parameter and strongly coupled complex system and an efficient and smooth operation is beneficial to the efficiency and stability of the PEMFC, and only under the optimal combination of temperature, stoich, oxidant, humidity and other parameters can the fuel cell achieve the best performance [34]. Operating temperature is the basic condition that determines the performance of fuel cells, and it not only affects the activity of the catalyst, but is also of great significance to the water management of the fuel cell. The gas stoich at the anode and cathode will also have a significant impact on the fuel cell performance. According to the existing research results [35,36,37,38], the current mainstream gas stoich of air is 2–3 and hydrogen is 1–2. Further analysis shows that the fuel cell will achieve its optimal performance under the conditions of high air stoich and medium hydrogen stoich, which shows that the comprehensive parameters of the fuel cell are optimal under this condition. In addition, too much gas will not only increase the difficulty of water management, but also cause waste. 

**Figure 4:**
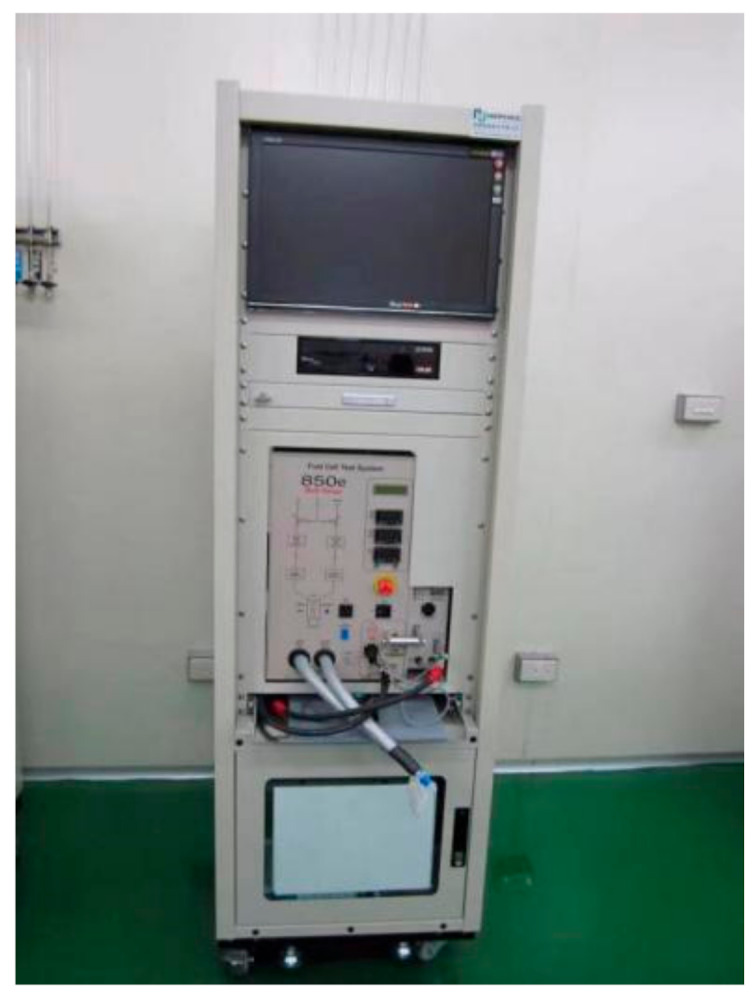
PEMFC test platform.

After installing the PEMFC on the test platform, we set the water temperature and gas heating temperature to 50 °C (as shown in Figure 4), and set a large gas flow to activate the PEMFC. When the performance was stable, we conducted the performance test, and set three cycles for each group of gas stoich, to avoid abnormal data. After the test, the temperature was adjusted to 60 °C and 70 °C, respectively, to analyze the performance of the PEMFC under different stoich conditions.

The whole test process mainly involves the following three variables: temperature, air stoich and hydrogen stoich, in which the temperature is constant. Therefore, the control variable method is used to systematically compare the performance differences of the PEMFC with different stoich conditions, and the specific parameter settings are shown in Table 1. Table 1 lists the current density of the PEMFC under different gas stoich conditions at 1.2 V, and with the increase in temperature and gas stoich, the PEMFC performance also gradually increases. This is because the increase in temperature can enhance the activation of the catalyst, and with the increase in gas, hydrogen and oxygen diffuse more fully in the PEMFC, and the two work together to improve the performance of the PEMFC. In addition, the addition of S_i_O_2_ effectively alleviates the problem of water loss caused by high gas flow, which indicates that the addition of S_i_O_2_ improves the water management ability of the fuel cell.

**Figure 5:**
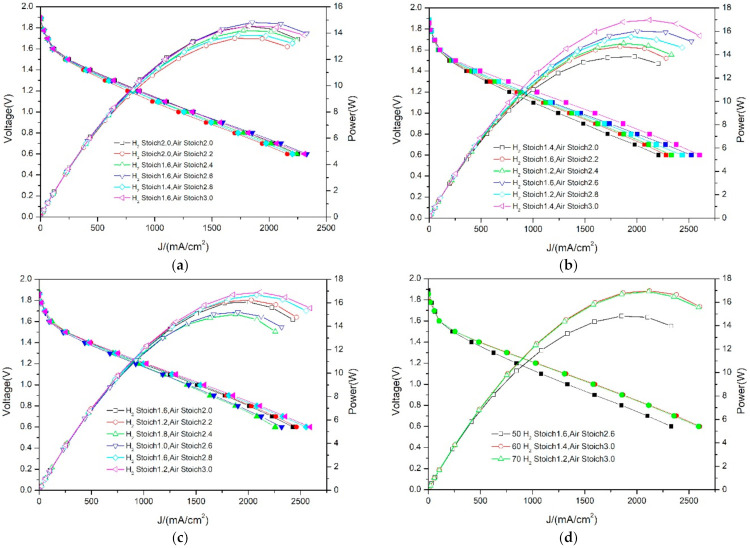
Comparison of PEMFC performance at different temperatures—air. (**a**) Optimal performance curve at 50 °C with different stoich. (**b**) Optimal performance curve at 60 °C with different stoich. (**c**) Optimal performance curve at 70 °C with different stoich. (**d**) Optimal performance curve at different temperatures.

From the perspective of research, the stability of the PEMFC under fixed voltage is important, but the performance needs to be further analyzed by cyclic voltammetry. In order to further compare the performance differences under different gas stoich conditions, the six best performance curves under three temperature conditions are drawn in the same figure (Figure 5a–c), and the three best performance curves are drawn in another figure (Figure 5d). Firstly, it was found from the test curve that the overall performance of the PEMFC was normal, and with the gradual decrease in voltage, the current and power increased gradually. Secondly, with the increase in temperature, the PEMFC performance also rose, but there was little difference between 60 °C and 70 °C, which indicates that blindly increasing the temperature cannot continuously improve the performance. At present, the mainstream operating temperature of a low-temperature fuel cell is 50–80 °C [31,37,39]. With the progress of technology, increasing the temperature is not the only means of improving the performance, and factors such as MEA characteristics and channel design will give full play to the fuel cell performance. The MEA used in this study can achieve the best performance, without raising the temperature to 70 °C. Finally, it is found from Figure 5d that the PEMFC can achieve the best performance under high air stoich and medium hydrogen stoich (3.0–1.4/3.0–1.6), which shows that it is not the hydrogen concentration that limits the performance, but the oxygen concentration in the air. If the hydrogen stoich continues to increase at the maximum air stoich, the water management inside the PEMFC will be affected, resulting in a decline in performance. The above analysis shows that the low oxygen concentration in air greatly limits the further improvement of PEMFC performance. Therefore, this paper used oxygen instead of air as a new oxidant, to study the performance difference between them.

### 3.3. Differences in PEMFC Performance at Different Temperatures—Oxygen

This section does not change the stoich of hydrogen, but replaces the oxidant from air with oxygen. A reasonable oxidant can not only improve the performance, but also effectively prolong the life, of a fuel cell. At present, there is little research on oxidants, and it is mainly focused on the field of performance improvement [16,35]. The results show that the use of oxygen greatly improves the performance of a fuel cell. In this study, the air stoich is increased from 2.0 times to 3.0 times, and the oxygen content in the air is 21%. In order to ensure the rationality of the comparison, the oxygen is also set to increase from 2.0 times stoich to 3.0 times stoich (0.2 times stoich/time). It can be seen from previous experiments that when the oxidant is air, the amount of gas required to generate 1A current per unit area is 17.5 mL/min. Therefore, after changing the oxidant to oxygen, the amount of gas required in theory is 3.5 mL/min, which shows that when the air and oxygen stoichs are the same, the absolute value of oxygen content is the same, but the concentration is different. Similar to Section 3.2, Table 2 lists the current density (1.2 V) of the PEMFC under different gas stoichs, under oxygen conditions. The overall condition is similar to that of air, that is, with the increase in temperature and gas stoich, the performance improves greatly, which indicates that high temperature and gas flow can effectively improve the performance of PEMFC under oxygen conditions.

**Figure 6:**
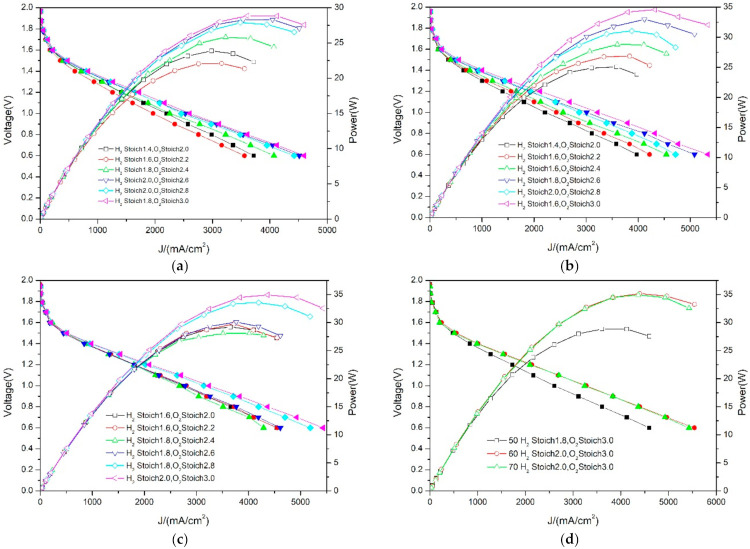
Comparison of PEMFC performance at different temperatures—oxygen. (**a**) Optimal performance curve at 50 °C with different stoich. (**b**) Optimal performance curve at 60 °C with different stoich. (**c**) Optimal performance curve at 70 °C with different stoich. (**d**) Optimal performance curve at different temperatures.

Because the decrease in gas flow under oxygen will inevitably affect its diffusion speed in the PEMFC, this paper set out to analyze the specific performance of the PEMFC through cyclic voltammetry curves. Figure 6 lists the six groups of voltammetry curves and optimal performance curves, with the best performance at different temperatures. It was found that the performance difference between PEMFC with a different stoich in the oxygen condition is significantly greater than that in the air condition. This is because the oxygen flow is much smaller than that of air, so the high stoich (3.0 times) can make the oxygen diffuse more fully inside the PEMFC, improving the performance of the PEMFC. As can be seen in Figure 6d, the best performance can be achieved only with high stoich hydrogen and oxygen, under oxygen conditions. This is because the use of oxygen greatly improves the performance of the catalyst and the sufficiency of the H_2–_O_2_ reaction. Therefore, with the increase in the stoich, the PEMFC performance continues to improve. As for the high oxidation of oxygen, the runner plate in this study is a carbon plate, so it has good corrosion resistance. From the voltametric curve, it is found that the performance is relatively stable on the whole, and there is no large performance fluctuation.

### 3.4. Comparison of Oxygen and Air Performance

**Figure 7:**
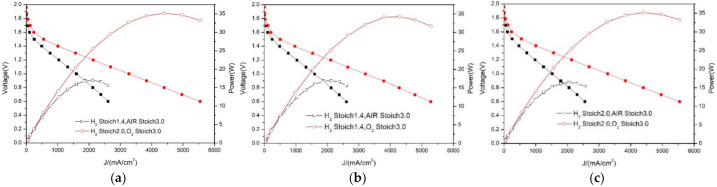
Comprehensive comparison of optimal performance of air–oxygen. (**a**) Comparison of air–oxygen optimal performance curves. (**b**) Comparison of air–oxygen performance under 1.4-H2 stoich. (**c**) Comparison of air–oxygen performance under maximum stoich.

Through the research and analysis in Section 3.2 and Section 3.3, it can be seen that the change of temperature and oxidant effectively improves the PEMFC performance. From the perspective of temperature, it was found that when the temperature increases from 50 °C to 60 °C, the performance improves significantly, but when the temperature further increases to 70 °C, the performance improvement is limited. Therefore, 60 °C is selected as the best working temperature in this paper. Figure 7 shows the optimal performance comparison of the PEMFC under air and oxygen, and the two curves in Figure 7a are the optimal curves under air and oxygen, respectively. Under 1.2 V, the performance is improved from 1038 mA/cm^2^ to 2134 mA/cm^2^, and the improvement rate is as high as 105.6%. In order to ensure the integrity of the comparison, Figure 7b,c shows the performance under the conditions of low and high stoich respectively, and they also achieved a performance improvement of 103% and 117.1%, respectively. The above analysis shows that the use of oxygen greatly improves the PEMFC performance, without affecting the stability, and has high research value.

## 4. Discussion

In the context of global energy conservation and emission reduction, the low pollution and high efficiency of fuel cells has been favored all over the world. However, from the perspective of commercial application, its economic attributes need to be systematically analyzed, so that fuel cells can really contribute to green development. It can be seen from the first section that the current research on the economics of fuel cells mainly focuses on cost calculation, but the universality of this calculation method is poor due to the inconsistent subsidy policies for new energy all over the world. Therefore, this research set out to calculate the issue of economics from the perspective of PEMFC power improvement, and the key link is oxygen production power. At present, mainstream industrial oxygen production technology is mainly divided into the following three types: cryogenic method, adsorption separation method and membrane separation method; the specific conditions are shown in Table 3. As seen in Table 3, the oxygen concentration produced by the cryogenic method is high, but there are problems, such as the high initial investment and large power consumption. The membrane separation method requires low investment and low power consumption, but the oxygen concentration is low. The adsorption separation method allows air to pass through the molecular sieve adsorption tower and use its selective adsorption of different molecules to realize the separation of oxygen, and this method has high concentration and high comprehensive cost performance. Therefore, the oxygen used in this study was prepared using this method.

As can be seen in Table 3, the electrical energy required to produce 1 Nm^3^ oxygen by the adsorption separation method is 0.32–0.37 kwh, so the average value is 0.35 kwh/Nm^3^. The following equation is based on the gas required at both ends of the PEMFC anode and cathode:

(7) P=I×λ×n×C

where *P* is gas flow (mL/min), *I* is electric current (A), *λ* is gas stoich, *n* is the number of PEMFC, *C* is amount of gas required per unit area (H_2_-7 mL/min, air-17.5 mL/min, O_2_-3.5 mL/min).

It can be seen from Figure 7 that the optimal performance under air is 1.2 V-10.38 A-12.5 W, and the optimal performance under oxygen conditions is 1.2 V-21.34 A-25.6 W. The gas input is H_2_-2 stoich and O_2_-3 stoich, so the amount of oxygen consumed is as follows:

(8) P=21.34×3×2×3.5=448.14 mL/min

The amount of oxygen consumed is 0.0268884 m^3^/h. The electrical energy consumed for the production of 1 Nm^3^ oxygen is 0.35 kWh, which is converted into power, that is, 350 W for 1 h. According to this principle, the O_2_ of 0.0268884 m^3^ need 9.41094 W (0.0268884 m^3^ × 350 W = 9.41094 W) for an hour, which is the unit of power required for the production of oxygen. Replacing air with oxygen, the performance of PEMFC is improved from 12.5 W to 25.6 W. After deducting 9.41094 W consumed by oxygen production, the net power increase is 3.68906 W.

On the other hand, the use of air as an oxidant inevitably leads to power consumption (gas filtration, pressurization, etc.), so the net power increase in oxygen compared with air is as follows:

(9) 3.68906 W/12.5 W=29.512%

With the expansion of production and the improvement of oxygen production technology, the use of oxygen can further expand the power gap between itself and air, which further highlights the rationality of the use of pure oxygen.

**Figure 8:**
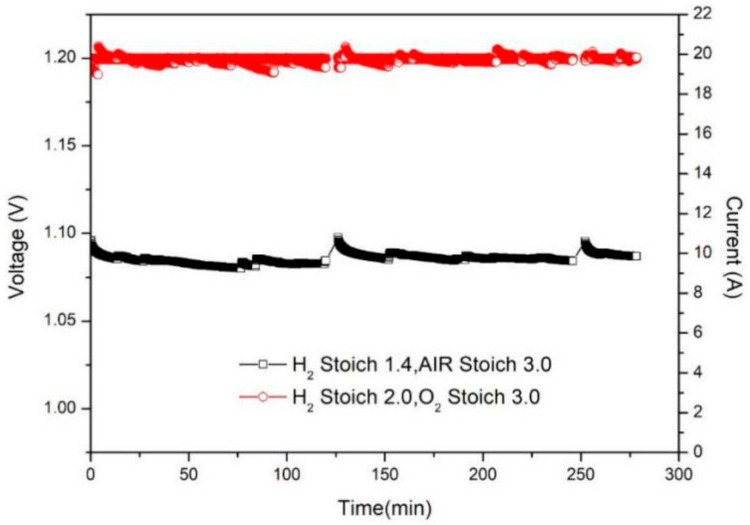
Air–oxygen performance difference under long-time load.

Large vehicles, represented by buses and tourist buses, are an important direction for the application of PEMFC, in the field of transportation vehicles. Large vehicles cannot only have high-power and large-volume fuel cell power systems, they must also drive smoothly, effectively protecting the stability of the power system performance. The typical characteristic of large-scale public transport is the desire for a long-term, stable operation, so it has high requirements for the continuous output of the power system. This paper studied the performance difference between air and oxygen PEMFC, under long-term constant voltage load. Figure 8 shows the performance difference of the PEMFC under long-time load, under the optimal stoich of air and oxygen at 60 °C. The overall operation of the PEMFC is stable, without large performance fluctuation, which shows that both air and oxygen are suitable for PEMFC. From the perspective of power improvement, the current of the PEMFC in air is stable at about 10 A, and oxygen is stable at about 20 A, and the performance improvement range is 100%, which shows that even for a long-time operation, the PEMFC with oxygen can still work stably, and its performance is much better than that of air.

From the perspective of economics, the average performance of air and oxygen under long-time load is 1.2 V-10 A-12 W and 1.2 V-20 A-24 W. Referring to formula (7), the oxygen consumption per minute is as follows:

(10) P=20×3×2×3.5=420 mL/min

According to the conversion, the oxygen consumption per hour is 0.0252 m^3^/h, and based on the power conversion relationship above, the power required to produce 0.0252 m^3^ of oxygen is 8.82 *W*. Therefore, the net increase in power is as follows:

(11) 24 W−12 W−8.82 W=3.18 W

This shows that under a long-time constant voltage operation, oxygen achieves a net power increase of 3.18 W/12 W = 26.5% compared with air.

## 5. Conclusions

PEMFC have the advantages of no pollution and high efficiency, so it has important research significance, given the background of global energy conservation, emission reduction and environment protection. In this paper, the performance and economics of PEMFC under different temperatures and oxidants were studied, and the water management of the cell was improved by adding SiO_2_ to MEA. The optimum operating temperature of the PEMFC was 60 °C, in both air and oxygen, and the overall performance was stable, without performance fluctuation under oxygen. Compared with air, the use of oxygen greatly improves the performance of the PEMFC by 105.6%, and the power is still improved by 29.5%, when the power consumption of oxygen production is removed. Under the condition of constant load for a long time, PEMFC performance is stable, and the use of oxygen achieves a net power increase of 26.5%.

## Figures and Tables

**Table 1:** Stoich setting of oxidant and reductant and performance under air (1.2 V).

	air	2.0(50–60–70)			2.2(50–60–70)			2.4(50–60–70)			2.6(50–60–70)			2.8(50–60–70)			3.0(50–60–70)		
H_2_																			
1.0		790	757	916	655	818	993	790	847	915	833	872	924	791	934	963	844	978	1015
1.2		827	764	927	698	835	1022	793	871	926	836	897	912	798	920	993	849	1006	1029
1.4		840	770	932	726	831	922	798	865	914	838	872	904	809	881	921	850	1038	1009
1.6		731	779	989	741	843	1007	807	850	914	846	919	924	817	891	999	864	1009	1014
1.8		754	778	998	756	845	988	813	849	947	839	961	917	826	881	1015	863	977	997
2.0		870	820	988	764	831	952	817	872	953	849	974	948	828	921	1002	872	983	1003

**Table 2:** Stoich setting of oxidant and reductant and performance under oxygen (1.2 V).

	O_2_	2.0(50–60–70)			2.2(50–60–70)			2.4(50–60–70)			2.6(50–60–70)			2.8(50–60–70)			3.0(50–60–70)		
H_2_																			
1.0		1304	1362	1594	1067	1455	1788	1342	1565	1637	1491	1658	1756	1439	1803	1878	1544	1926	2029
1.2		1324	1344	1650	1152	1469	1861	1372	1612	1685	1529	1731	1760	1492	1786	1956	1579	2012	2078
1.4		1411	1395	1696	1218	1521	1816	1397	1651	1674	1551	1727	1754	1538	1779	1953	1631	2107	2068
1.6		1228	1387	1820	1260	1559	1863	1437	1658	1737	1565	1837	1802	1576	1835	1999	1702	2118	2109
1.8		1236	1384	1757	1247	1546	1808	1422	1656	1799	1586	1911	1806	1612	1842	2020	1726	2100	2095
2.0		1410	1460	1739	1245	1505	1675	1430	1682	1753	1639	1958	1867	1649	1935	2054	1718	2134	2097

**Table 3:** Comparison of oxygen production cost under different processes.

Index	Cryogenic Method		Adsorption Separation Method	Membrane Separation Method
	High-Purity	Low-Purity		
Yield (m^3^/h)	50–100,000	1000–100,000	50–4000	25–15,000
Purity (%)	99.6	95.0	93.0	40.0
Pressure (MPa)	0.02–0.50	0.02–0.50	0.01	0.01
Energy consumption (KWh.m^−3^)	0.45–0.80	0.40–0.60	0.32–0.37	<0.30
Run-up time (h)	30	28	0.2	0.1
Product costs	middle	Slightly lower	Low	Low
Equipment investment	High	Slightly lower	Slightly lower	Low
Operability	Complex	Complex	Easy	Easy
Floor space	Big	Big	Middle	Small

## Data Availability

The data used to support the findings of this study are included within the article. All required models and parameters are listed in the article.
